# Changes in acromegaly comorbidities, treatment, and outcome over three decades: a nationwide cohort study

**DOI:** 10.3389/fendo.2024.1380436

**Published:** 2024-04-04

**Authors:** Christian Rosendal, Mai Christiansen Arlien-Søborg, Eigil Husted Nielsen, Marianne Skovsager Andersen, Claus Larsen Feltoft, Marianne Klose, Mikkel Andreassen, Niels Henrik Bruun, Jens Otto Lunde Jørgensen, Jakob Dal

**Affiliations:** ^1^ Department of Endocrinology, Aalborg University Hospital, Aalborg, Denmark; ^2^ Department of Endocrinology and Internal Medicine, Aarhus University Hospital, Aarhus, Denmark; ^3^ Department of Endocrinology, Odense University Hospital, Odense, Denmark; ^4^ Department of Medicine, Copenhagen University Hospital, Herlev, Denmark; ^5^ Department of Endocrinology, Gentofte Hospital, Herlev, Denmark; ^6^ Department of Endocrinology and Metabolism, Copenhagen University Hospital Rigshospitalet, Copenhagen, Denmark; ^7^ Research Data and Biostatistics, Aalborg University Hospital, Aalborg, Denmark; ^8^ Steno Diabetes Center North Jutland, Aalborg, Denmark

**Keywords:** cancer endocrinology, acromegaly, acromegaly and cancer, acromegaly treatment, pituitary adenomas, acromegaly comorbidities

## Abstract

**Objective:**

To study the time-dependent changes in disease features of Danish patients with acromegaly, including treatment modalities, biochemical outcome, and comorbidities, with a particular focus on cancer and mortality.

**Methods:**

Pertinent acromegaly-related variables were collected from 739 patients diagnosed since 1990. Data are presented across three decades (1990–1999, 2000–2009, and 2010–2021) based on the year of diagnosis or treatment initiation.

**Results:**

Adenoma size and insulin-like growth factor I (IGF-I) levels at diagnosis did not differ significantly between study periods. The risk of being diagnosed with diabetes, heart disease, sleep apnea, joint disease, and osteoporosis increased from the 1990s to the later decades, while the mortality risk declined to nearly half. The risk of cancer did not significantly change. Treatment changed toward the use of more medical therapy, and fewer patients underwent repeat surgeries or pituitary irradiation. A statistically significant increase in the proportion of patients achieving IGF-I normalization within 3–5 years was observed over time (69%, 83%, and 88%). The proportion of patients with three or more deficient pituitary hormones decreased significantly over time.

**Conclusion:**

Modern medical treatment regimens of acromegaly as well as increased awareness and improved diagnostics for its comorbidities have led to better disease control, fewer patients with severe hypopituitarism, and declining mortality in the Danish cohort of acromegaly patients. The risk of cancer did not increase over the study period.

## Introduction

Acromegaly is a systemic disease caused by increased growth hormone (GH) secretion, predominantly due to a pituitary adenoma (1). The disease is rare with incidence rates of 3.1 to 5.3 cases/million/year (2–8) and a reported prevalence (per million) in newer studies ranging from 83 to 137 (3, 4, 6, 9, 10). The incidence appears to increase, and a shift toward a milder disease phenotype has been suggested (4, 11–13). Disease onset is slow and insidious, and a diagnostic delay of 4–10 years is not uncommon (1, 14), which carries a risk of irreversible complications (1).

Acromegaly is associated with a risk of numerous complications from multiple organ systems, including hypertension, type II diabetes, heart disease, osteoarthritis, sleep apnea, and osteopathy (1). Thus, the treatment of acromegaly entails not only treating the pituitary tumor and the resulting growth hormone hypersecretion but also management of comorbidities (15). In some patients, this also includes replacement therapy of hypopituitarism, which may be secondary to the pituitary tumor or its treatment. Like in other pituitary tumors, suprasellar extension of the adenoma may result in visual disturbances or headaches. The risk of cancer in acromegaly is still being debated; an increased risk of thyroid and colorectal cancers has consistently been reported, although surveillance bias may be an issue (15, 16). Recent meta-analyses have reported a slightly increased overall cancer risk (16, 17), but cancer-related mortality seems to be comparable to the background population (18).

When possible, the first-line treatment of acromegaly is surgical removal of the pituitary adenoma (19), taking into consideration adenoma size, localization, and degree of invasiveness. Considerable advances in medical treatment have been made since the 1970s when dopaminergic agonists (DAs) were introduced. The first-generation somatostatin analogs (SSAs) were introduced in the 1990s and have gained a central position in the medical treatment of acromegaly, leaving only a secondary role for DAs in many countries. Lastly, second-generation SSAs and GH receptor antagonists (GHRAs) have now been available for more than a decade. The availability of medical alternatives has reduced the use of pituitary tumor irradiation and, in turn, reduced irradiation-induced adverse effects such as secondary intracranial tumors, cerebrovascular disease, and hypopituitarism (20). The diverse treatment options allow for a more personalized and multimodal treatment approach, leading to disease control in ~60%–90% of patients (4, 6, 21, 22).

This study aimed to map the changing landscape of acromegaly epidemiology, treatment, comorbidities, and mortality as a function of time, using the nationwide Danish Acro_DEN_ cohort.

## Materials and methods

### Study population

The study population comprised the entire cumulative population of Danish patients with acromegaly diagnosed from 1990 to 2021. The Danish national healthcare system provides tax-supported medical care free of charge for the patients, and since 1977, all outpatient clinic visits have been recorded in the Danish National Patient Registry (DNPR) and coded using the International Classification of Diseases (ICD)-8 and ICD-10 codes. Using this registry and the CPR number (a unique personal identification number assigned to all Danish citizens upon birth or immigration) enables the identification of all incident and prevalent patients with acromegaly dating back to 1977: the Acro_DEN_ cohort. Patients for the present study were identified using the ICD-8 and ICD-10 codes for acromegaly, and the diagnosis was subsequently validated by manual chart review, including biochemical confirmation of acromegaly, a method previously described by this group (23, 24). Patients with pertinent signs and symptoms of acromegaly and/or elevated insulin-like growth factor I (IGF-I) and/or lack of glucose-induced GH suppression were included. Patients with co-secretion of prolactin were also included, whereas false-positive cases (i.e., patients with an ICD diagnosis code of acromegaly, but no biochemical confirmation of acromegaly) were excluded.

### Data collection

The following acromegaly-related clinical variables were retrieved from the patient charts: pituitary adenoma size, baseline fasting and nadir GH measurements, IGF-I measurements at the time of diagnosis and follow-up (3–5 years after diagnosis), acromegaly-specific treatment, comorbidities, and long-term hormone replacement therapies. Dates of acromegaly diagnosis, transsphenoidal surgeries, initiation of medical treatment (SSAs, GHRAs, DAs, and hormone replacement therapy), pituitary irradiation, and follow-up were also collected. Data on comorbidities (hypertension (treatment with antihypertensive agents), X-ray-verified arthropathy/osteoarthritis (joint disease), polysomnography-verified obstructive sleep apnea, type II diabetes mellitus [elevated glycated hemoglobin (HbA1c) or treatment with antidiabetic agents], osteoporosis (T-score ≤−2.5 or treatment with antiresorptive agents), heart disease (history of ischemic heart disease, valvular heart disease, congestive heart failure, or arrhythmias), and cancer (excluding non-melanoma skin cancers), as well as date of diagnosis for each condition, were retrieved from medical records.

Different IGF-I assays were used during the observation period. As the reference values for IGF-I depend on age and sex, reference limits for each sample were collected in order to calculate the relative IGF-I increase relative to the upper reference limit [times upper limit of normal (×ULN)].

The study was approved by the North Denmark Region (approval no. 2021-004763 and 2021-173) in accordance with the regulations set forth by the Danish Data Protection Agency and the Danish Health Act §46-48. All data were entered into a Research Electronic Data Capture (REDCap) database and stored on secure servers belonging to the North Denmark Region.

### Statistical analysis

Normally distributed data are presented as mean ± standard deviation (SD) and compared across study periods using ANOVA. Non-normally distributed data are presented as median and interquartile range (IQR), and differences across study periods were analyzed using the Kruskal–Wallis test.

Categorical data were analyzed using Pearson’s chi^2^ test. Binary regression was used to compare binary data between study periods. Cox regression analysis, the Kaplan–Meier plots, and log-rank tests were used to analyze time-to-event data, with patients starting observation at the date of birth, analysis time beginning at the time of diagnosis of acromegaly, and ending at the time of diagnosis of a given comorbidity, death, or end-of-study (December 31, 2021). A flexible parametric equations survival model served to analyze the mortality rate curve assuming non-proportional hazards. p < 0.05 was considered statistically significant.

Statistical analysis was performed using Stata v. 17.0 for Mac and Stata v. 18.0 (StataCorp. 2021. *Stata Statistical Software: Release 17 and 18*. College Station, TX: StataCorp LLC).

Graphs were composed in Excel for Mac (v. 16.78.3, Microsoft Corporation) and Stata v. 18.

## Results

### Patient characteristics

A total of 736 incident patients diagnosed during the period 1990–2021 were included, of which 609 patients were alive at the end of the study (December 31, 2021). Data are presented across three study periods: 1990–1999 (201 incident cases), 2000–2009 (223 incident cases), and 2010–2021 (312 incident cases) (**Table 1**). The mean age at acromegaly diagnosis was 49.3 ± 14.9 years and did not change significantly across study periods. In the study period, six patients were under the age of 18 at diagnosis (mean 12.5 years; range, 6.3–17.7): three patients in the 2000s and 2010–2021 periods. Sex composition was constant and even with 51% female. Adenoma size remained constant throughout the study period (median 16 mm, IQR 10–23 mm), and the proportion of macroadenomas was 73%–79% and did not differ significantly between the three study periods. IGF-I levels (×ULN) at diagnosis remained constant [1990s, 3.1 (±1.6); 2000s, 2.8 (±1.1); 2010–2021, 2.9 (±1.2)]. Both fasting and nadir GH levels decreased significantly over time (p = 0.007 and p < 0.001, respectively). Data on prolactin levels were available in the latest study period, where 19% of patients had co-secretion of GH and prolactin.

**Table 1:** Baseline characteristics of acromegaly patients diagnosed between 1990 and 2021.

	Total (n = 736)	1990–1999 (n = 201)	2000–2009 (n = 223)	2010–2021 (n = 312)	p
Age at diagnosis (years), mean (SD)	49.3 (14.9)	48.1 (13.9)	48.5 (15.3)	50.7 (15.3)	NS
Gender, n and % female	374 (51%)	93 (46%)	116 (52%)	165 (53%)	NS
Adenoma size (mm), median (IQR)	16 (10–23)	15 (9–23)	17 (10–25)	15 (10–22)	NS
Macroadenomas, n (%)	414 (77%)	86 (73%)	125 (77%)	203 (79%)	NS
IGF-1 × ULN at diagnosis, mean (SD)	2.9 (1.2)	3.1 (1.6)	2.8 (1.1)	2.9 (1.2)	NS
Fasting GH (μg/L), median (IQR)	8.1 (3.9–17.6)	10.2 (4.7–20.6)	8.9 (4.4–21.0)	7.4 (3.4–13.0)	0.007
Nadir GH (μg/L), median (IQR)	7.2 (3.3–17.6)	10.0 (4.4–23.7)	8.2 (3.5–18.6)	5.7 (2.5–13.0)	<0.001

SD, standard deviation; IQR, interquartile range; × ULN, times upper limit of normal; NS, not significant.

### Risk of acromegaly-related comorbidities and mortality

**Figure 1:**
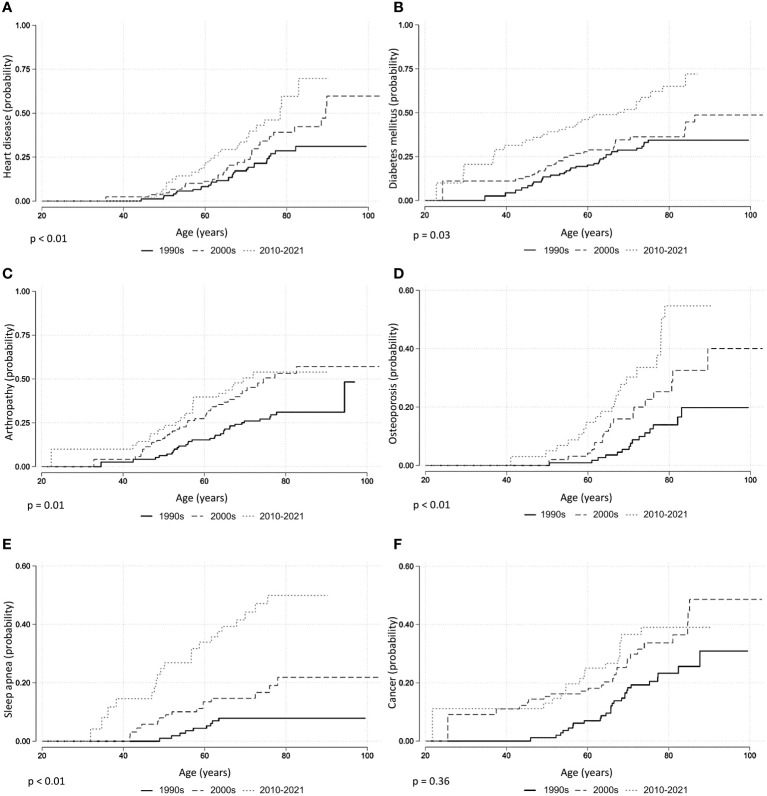
Comorbidities in acromegaly, Kaplan–Meier curves. **(A)** Heart disease. **(B)** Diabetes. **(C)** Arthropathy. **(D)** Osteoporosis. **(E)** Sleep apnea. **(F)** Cancer. p-Values from log-rank test.

**Figure 2:**
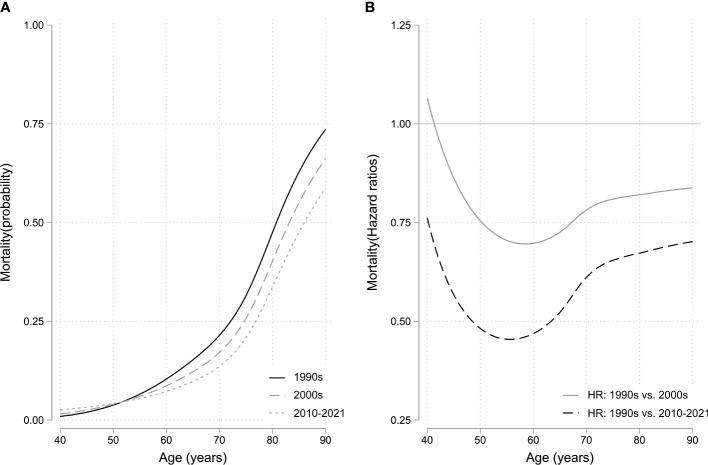
**(A)** Mortality rate curve using flexible parametric survival model with non-proportional hazards. **(B)** Hazard ratios as a function of biological age using diagnosis decade 1990–1999 as reference. HR, hazard ratio.

Across the entire cohort, 358 (49%) patients were diagnosed with hypertension, 231 (31%) with joint disease, 191 (26%) with type II diabetes, 138 (19%) with heart disease, 118 (16%) with sleep apnea, and 80 (11%) with osteoporosis. Between the patients diagnosed with acromegaly in the 1990s vs. the 2000s, there was a statistically significant increased risk of being diagnosed with joint disease (HR 1.96 [1.21; 3.18]), osteoporosis (HR 2.21 [1.13; 4.32]), and sleep apnea (HR 2.34 [1.02; 5.37]) (**Table 2**; **Figure 1**). Between patients diagnosed in the 1990s vs. 2010–2021, there was a statistically significant increased risk of being diagnosed with heart disease (HR 2.48 [1.44; 4.28]), diabetes (HR 1.87 [1.12; 3.13]), joint disease (HR 1.88 [1.05; 3.35]), osteoporosis (HR 4.12 [2.12; 8.02]), and sleep apnea HR (6.38 [2.89; 14.08]). The mortality risk significantly decreased from the 1990s to the 2010–2021 period (HR 0.55 [0.30; 0.99], **Figure 2**), whereas there was no difference in the risk of receiving a cancer diagnosis between the first study period and the last two decades (**Table 2**; **Figure 1**).

**Table 2:** Comorbidities, initiation of any long-term hormone replacement therapy and mortality; Hazard ratios (95% confidence interval).

	1990s vs. 2000s	1990s vs. 2010–2021
Heart disease	1.52 (0.91; 2.55)	2.48* (1.44; 4.28)
Diabetes	1.06 (0.63; 1.78)	1.87* (1.12; 3.13)
Joint disease	1.96* (1.21; 3.18)	1.88* (1.05; 3.35)
Osteoporosis	2.21* (1.13; 4.32)	4.12* (2.12; 8.02)
Sleep apnea	2.34* (1.02; 5.37)	6.38* (2.89; 14.08)
Cancer	1.30 (0.75; 2.26)	1.55 (0.83; 2.91)
Hypopituitarism	1.31 (0.98; 1.76)	1.14 (0.85; 1.55)
Mortality	0.88 (0.59; 1.29)	0.55* (0.30; 0.99)

*Statistical significance, Cox regression model, and log-rank test with diagnosis decade 1990–1999 as reference.

### Acromegaly and cancer

A total of 72 patients (10%) with acromegaly were diagnosed with cancer after the time of acromegaly diagnosis (**Table 3**): 31 patients (1990–1999), 25 patients (2000–2009), and 16 patients (2010–2021). In the subgroup of patients diagnosed with cancer, the mean age at cancer diagnosis was constant at 63 years (±12.9 years, p = 0.20). A significant decrease in time from acromegaly diagnosis to cancer diagnosis was observed, declining from 18.2 years (±8.7 years) in the first study period to 4.6 years (±2.6 years) in the last (p < 0.001), whereas the mean age at acromegaly diagnosis increased, although not with statistical significance, in each decade from 47.4 (±10.1 years) in the 1990s to 54.1 years (±11.9 years) in the 2010–2021 period (p = 0.10). There was no difference across study periods regarding pituitary adenoma size, the proportion of macroadenomas, IGF-I × ULN at diagnosis, or the proportion of patients achieving IGF-I control at 3–5 years or the latest follow-up in acromegaly patients with a cancer diagnosis.

**Table 3:** Characteristics of acromegaly patients with cancer comorbidity (including patients who were diagnosed with cancer at any point after the diagnosis of acromegaly).

	Total (n = 72)	1990s (n = 31)	2000s (n = 25)	2010–2021 (n = 16)	p
Age at acromegaly diagnosis (years), mean (SD)	50.9 (12.2)	47.4 (10.1)	53.2 (14.0)	54.1 (11.9)	NS
Gender, n and % female	38 (53%)	11 (35%)	18 (72%)	9 (56%)	0.023
Age at cancer diagnosis (years), mean (SD)	63.0 (12.9)	65.8 (10.2)	62.6 (15.6)	58.7 (12.2)	NS
Time from acromegaly diagnosis to cancer diagnosis (years), mean (SD)	12.0 (8.6)	18.2 (8.7)	9.5 (5.5)	4.6 (2.6)	<0.001
Adenoma size (mm), median (IQR)	15 (9-20)	10 (8-17)	15 (10-20)	16 (12-23)	NS
Macroadenomas, n (%)	34 (69.4%)	6 (46.2%)	16 (76.2%)	12 (80.0%)	NS
IGF-I × ULN at diagnosis, mean (SD)	2.9 (1.3)	2.8 (1.1)	2.8 (1.5)	3.4 (1.1)	NS
IGF-I × ULN ≤ 1.2 at 3–5 years of follow-up, n (%)	41 (71.9%)	10 (52.6%)	18 (78.3%)	13 (86.7%)	NS
IGF-I × ULN ≤ 1.2 at latest follow-up, n (%)	56 (87.5%)	25 (96.2%)	20 (87.0%)	11 (73.3%)	NS

SD, standard deviation; IQR, interquartile range; ×ULN, times upper limit of normal; NS, not significant.

### Treatment modalities and biochemical disease control

**Figure 3:**
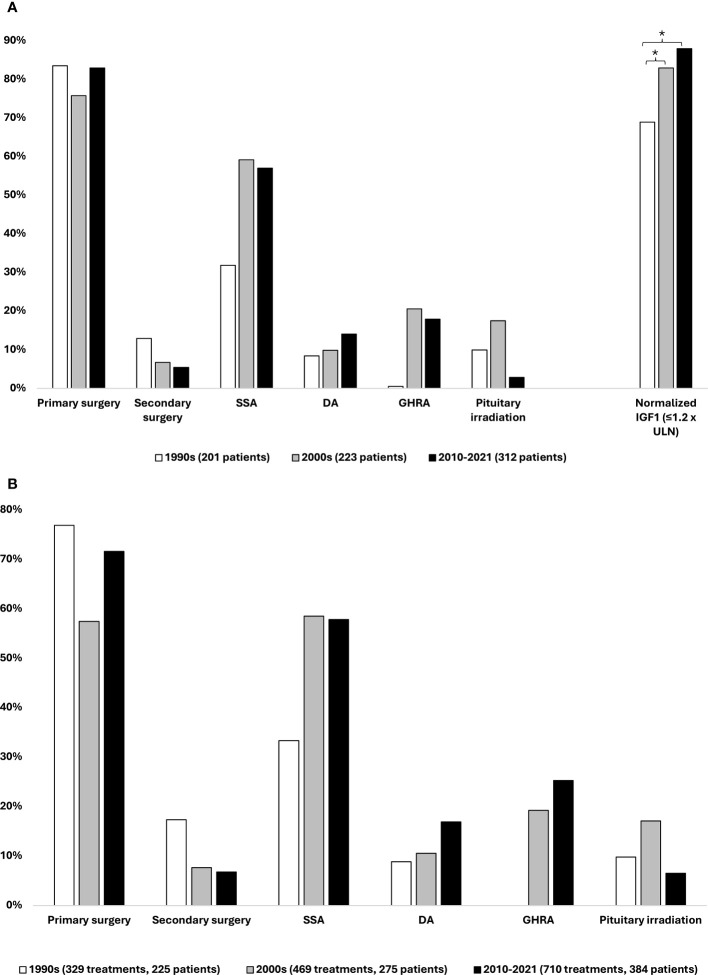
Acromegaly treatment. **(A)** Distribution of treatment modalities performed or initiated within 5 years of acromegaly diagnosis on incident patients from each study decade, 1990–2021. **(B)** Distribution of treatment modalities performed or initiated on prevalent patients in each study decade, 1990–2021. The same patient may have received more than one type of treatment; thus, the percentages add up to more than 100. * Statistically significant difference between study periods, binary regression with diagnosis decade 1990–1999 as reference.

The distribution of treatment modalities of the *incident* patients from each study period was based on treatments performed or initiated within 5 years of diagnosis (**Figure 3A**). Furthermore, the corresponding numbers for the *prevalent* patients in each decade are presented in **Figure 3B** and were based on the total number of surgical or medical treatments performed or initiated on the cumulated number of prevalent patients with acromegaly receiving treatment in a given decade (1990s, n = 329 treatments/225 patients; 2000s, n = 469 treatments/275 patients; 2010–2021, n = 710 treatments/384 patients).

### Incident cases of acromegaly

Within 5 years of diagnosis of acromegaly, 76%–83% of the incident patients received primary pituitary surgery, whereas the proportion of patients requiring secondary surgery within 5 years of diagnosis decreased from 13% in the 1990s to 7% in the 2000s and 5% in the period 2010–2021.

Following the introduction of SSAs in the 1990s, treatment with these drugs was initiated on 32%, 59%, and 57% of the incident patients over the three study periods. Since its approval in 2014 and until the end of 2021, the second-generation SSA pasireotide constituted 10% (14/141) of initiated treatments with SSAs. The use of dopamine agonists exhibited an increase in use, with 9%–10% of patients initiating treatment with these agents in the 1990s and 2000s and 14% in the latest study period. Further analysis revealed that 65%–77% of DA treatments were initiated as adjunctive therapy to an SSA. The GHRA pegvisomant was introduced in Denmark in 2002, and as such, only 0.5% of patients diagnosed in the 1990s (n = 1) initiated treatment with a GHRA within 5 years of diagnosis, whereas this number was 21% and 18% for the middle and last study decades, respectively. Radiotherapy was rarely utilized, with 9% of patients being treated with this modality within 5 years of diagnosis, across the entire cohort. Fractionated stereotactic radiotherapy was most widely used, whereas proton therapy was applied more rarely and only in the later study periods.

The proportion of incident patients achieving normalized IGF-I values (defined as IGF-I ≤ 1.2 × ULN) at follow-up 3–5 years after acromegaly diagnosis (mean 4.2 ± 0.9 years) significantly increased over time (**Figure 3A**). An increase in the proportion of patients with normalized IGF-I levels was observed from 69% of patients diagnosed in the 1990s to 83% of patients diagnosed in the 2000s (p = 0.005) and 88% of those diagnosed in the 2010–2021 period (p < 0.001). This corresponds to a 21% (RR 1.21 [1.06;1.39], p = 0.005) and 27% (RR 1.27 [1.12;1.45], p < 0.001) increase in the proportion of patients achieving IGF-I control between the 1990s to the 2000s and 2010–2021 period, respectively.

### Prevalent patients with acromegaly

Primary pituitary surgery was the treatment modality most often used across the study periods, being performed in 58%–77% of treated patients and surpassed by SSAs only in the 2000s. Use of repeat surgery declined throughout the study period, from 17% in the 1990s to 7% in the latest study period. SSAs were used in 33%–56% of treated patients. The use of dopaminergic agonists, GHRAs, and pituitary irradiation therapy followed similar trends as described above. Of treatments with pegvisomant, 95% (142/150) were initiated as adjunctive therapy to an SSA.

The total number of treatments in each decade increased by 42% and 115% from the 1990s to the 2000s and 2010–2021 period, respectively. Based on this and the number of treated patients in each study period, the number of treatments per patient increased from 1.46 to 1.71 and 1.85 over the three study periods: a 26% increase from the first to the last study period.

### Risk of hypopituitarism

**Figure 4:**
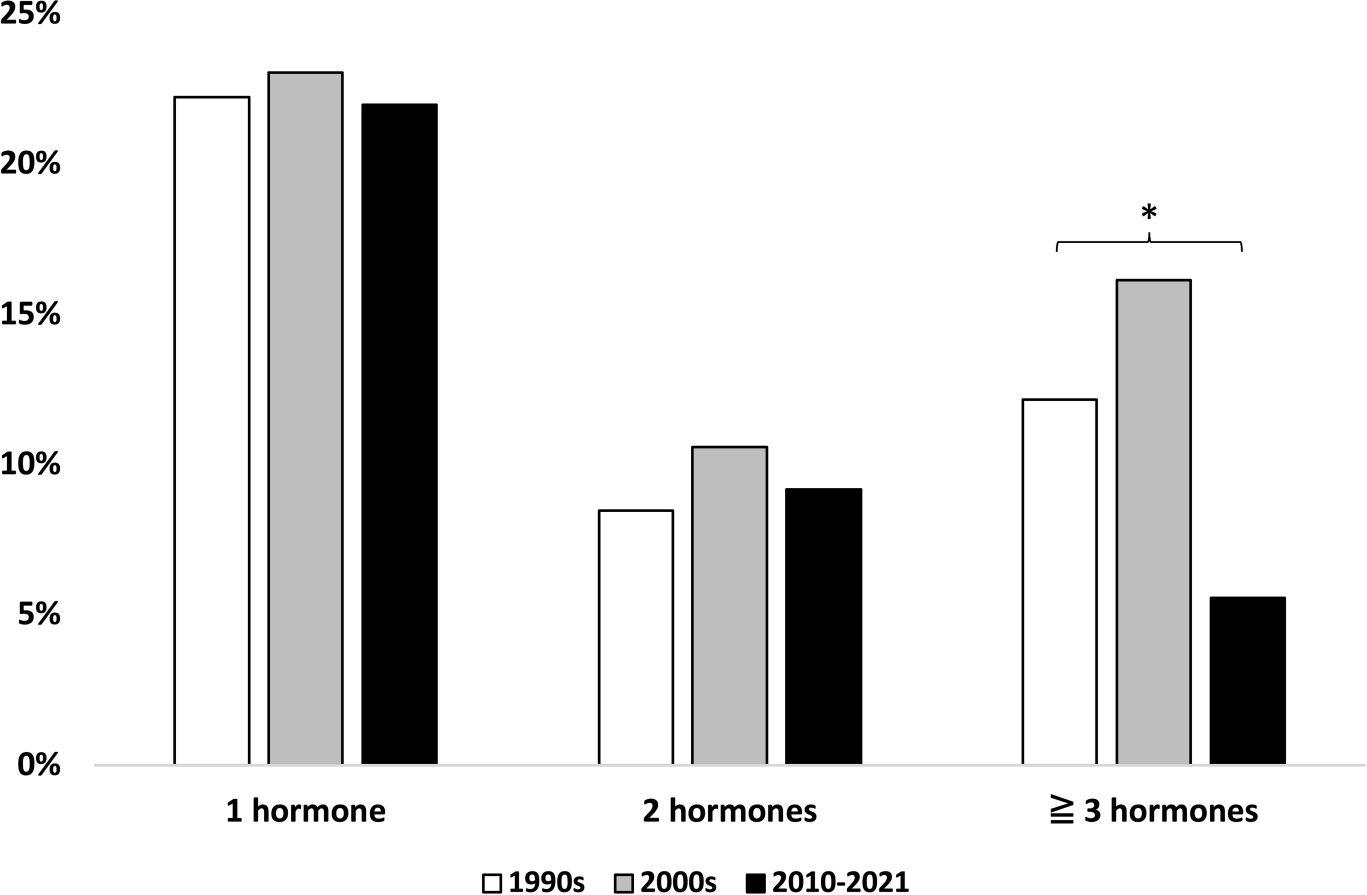
Number of deficient pituitary hormones across study periods. * Statistically significant difference, chi^2^ test.

Evaluation of hypopituitarism is based on the initiation of long-term pituitary hormone replacement therapy. Across the entire cohort, 301 patients (41%) received long-term replacement of one or more pituitary hormones. A total of 195 patients (27%) received levothyroxine, 149 (20%) hydrocortisone, 145 (20%) sex hormone, and 41 (6%) vasopressin; 12 (2%) were treated with growth hormone substitution. Several patients received multiple hormone substitutions. The proportion of patients with one (22%–23%) or two (9%–11%) deficient pituitary hormones did not differ markedly between the decades, whereas the proportion of patients with three or more deficient pituitary hormones was lower in the latest study decade [16% (1990s) vs. 12% (2000s) and 6% (2010–2021), p = 0.045 (**Figure 4**)].

## Discussion

This study presents clinical and biochemical data on our nationwide Acro_DEN_ cohort over a three-decade period. The main findings were an increasing risk of acromegalic comorbidities over time, an increasing proportion of patients achieving disease control, decreasing mortality, and a declining proportion of patients with hypopituitarism. Cancer risk was unchanged but with a shorter time from acromegaly diagnosis to cancer diagnosis.

Acromegaly is associated with a wide range of comorbidities, and the distribution of these comorbidities in our cohort mirrors that of similar cohorts (21, 25, 26). We observed a time-dependent increased risk of heart disease, osteoporosis, diabetes, joint disease, and sleep apnea, possibly reflecting increased physician awareness and screening for these complications (15). This is in line with the decreasing cardiovascular mortality reported in acromegaly (24, 27), indicating an increased focus on the treatment of comorbidities, rather than an increased risk of comorbidities. Indeed, a mortality rate comparable to the general population has previously been reported from the first iteration of this cohort (HR 1.3 [1.0; 1.7] (24)). The occurrence of sleep apnea increased substantially over time, which may be attributable to increased awareness and improved treatment options since the condition has garnered increasing focus since the 1990s (15, 28, 29). The risk of being diagnosed with osteoporosis also markedly increased from the 1990s to the latest study period, presumably due to the increasing focus on acromegaly and bone disease in recent years (30, 31). Treatment guidelines now recommend screening with bone densitometry and spine imaging in all patients with acromegaly (15), whereas earlier guidelines recommended screening only in the setting of concomitant hypogonadism (28). Novel treatment options for osteoporosis have also emerged, although their effect on acromegalic bone disease has only been sparsely studied (32).

Interestingly, cancer was the only comorbidity for which the risk did not significantly increase over the three decades. Cancer was diagnosed in a total of 72 patients, corresponding to 10% of our study population, which is similar to previous publications (6, 21, 26). The modest cancer risk in our cohort aligns well with findings from recent meta-analyses, where population-based studies displayed lower cancer rates as compared to single-center studies and only modestly increased overall cancer risk [standardized incidence ratio (SIR), 1.45 [1.20; 1.75] and 1.5 [1.2; 1.8], respectively] (16, 17). In these studies, colorectal, thyroid, breast, and urinary tract cancers exhibited increased incidence in patients with acromegaly, although the overall cancer risk was only slightly increased. As per the Danish guidelines for the treatment of acromegaly, patients are recommended to follow the national cancer screening programs, e.g., mammography screening and fecal occult blood tests (33). However, as patients are followed up closely at highly specialized centers, symptoms suggestive of cancer are likely managed more closely. This is supported by the fact that cancer-specific mortality is not increased, as previously reported from this cohort (16) and observed in other comparable cohorts (6, 18, 34). The incidence of cancer was not found to be increasing in our cohort across the study periods, despite GH excess being a well-established risk factor for the development of cancer. Possible explanations may include decreasing diagnostic delay, as has been reported from other cohorts (14), or improvements in disease control. Lastly, disease characteristics have been described as becoming milder (13), possibly providing the basis for the stable cancer risk. In the present study, however, we did not observe a statistically significant decline in IGF-I levels at diagnosis, whereas we did find a significant decrease in both fasting and nadir GH levels at diagnosis. This should be interpreted in the context of changing assays over the study period, i.e., a shift from poly- to monoclonal antibodies and increasingly sensitive assays, rather than declining levels of GH over time.

In our cohort, the risk of cancer was not associated with either uncontrolled acromegaly or a specific treatment of acromegaly. Among patients with acromegaly and cancer, the mean age at cancer diagnosis was 63 years, which aligns well with data reported in a recent meta-analysis on acromegaly and cancer (16), but is slightly lower than that of a large cohort of Danish cancer patients, recently reported to be 67 years (35). As was the case for the entire cohort, age at acromegaly diagnosis in the cancer subgroup tended to increase across the study periods, while the age at cancer diagnoses was stable, resulting in a decline in time from acromegaly to cancer diagnosis. This can most likely be attributed to the most recently diagnosed patients having shorter follow-up periods but may also suggest an increasing awareness similar to what was observed regarding other comorbidities.

As seen across all three study periods, primary pituitary surgery continues to be the mainstay of acromegaly treatment, being used to treat 76%–83% of incident patients in our cohort, which is in keeping with other similar works (4, 7, 22). However, the management of acromegaly has become increasingly complex due to the availability of newer pharmacological treatment options, and physicians now have a broader range of therapeutic options at hand. As such, medical therapy has gained increasing use as both primary and secondary treatment, in accordance with other surveys (21, 22, 25, 26). As reported in other cohorts (7, 21, 22, 36), the treatment has become more personalized, combining several therapies to treat the individual patient. This is also reflected in the absolute number of treatments having more than doubled and the 26% increase in the number of treatments performed per patient in each study decade, in turn resulting in an increasing number of contacts with the healthcare system. The more personalized approach enables a larger proportion of patients in our cohort to achieve biochemical disease control, reaching 88% at 3–5 years of follow-up in patients from the latest diagnosis period. A similar proportion of patients achieving biochemical disease control was reported from recent French (21), Swedish (22), and American series (36), while it is somewhat higher than what is reported from other national registries [37%–76%, mean 61% (37)]. The increasing use of medical therapy in our cohort evidently reduced the need for secondary surgery or pituitary irradiation; this shift away from repeat surgery and pituitary irradiation may explain the decreasing proportion of patients developing hypopituitarism.

We observed a decrease in mortality from the first to last study periods, where it was reduced by almost half. This can likely be attributed to improved disease control, as well as increased focus on, and more effective treatment of comorbidities. The decrease in mortality is in keeping with a meta-analysis from 2018, where the standardized mortality ratio in acromegaly was found to be lower in more recent publications, as compared to earlier studies, and causes of death were shifting from cardiovascular to neoplastic (27). However, cancer-related mortality has not been found to be increased when compared to the background population (13). Moreover, the types of cancer observed in acromegaly patients seem to have become more diverse in recent surveys (21, 27), which has been attributed to the increasing life expectancy of patients with acromegaly, exposing them to cancers typically associated with aging and environmental factors (16, 18, 27).

A strength of the present study lies in the fact that, upon diagnosis, all patients with acromegaly in Denmark are treated and monitored in one of five specialized pituitary centers, ensuring uniform treatment across the country. Furthermore, free and equal access to medical care for all Danish citizens through the public healthcare system minimizes cost- or insurance-related barriers to medical care. Finally, the Danish registries based on unique ID numbers for all citizens allow virtually complete follow-up. Lastly, all cases were validated by biochemical confirmation of the acromegaly diagnosis.

A potential limitation of the present study is that medical charts may not be exhaustive as regards the amount or type of information that was collected retrospectively. As this study focused on within-cohort changes over time, there is no comparison group. Furthermore, GH and IGF-I assays changed during the study period. This was overcome by presenting data as relative measures, such as times upper limit of normal, rather than the absolute values. Finally, the data presented in the present study did not distinguish between the type or severity of cancer nor the cause of death.

## Conclusion

In conclusion, modern, individualized acromegaly treatment has led to an increasing proportion of patients achieving disease control, as well as a smaller proportion of patients with severe hypopituitarism. This, in conjunction with an increasing focus on the detection and treatment of comorbidities, has resulted in decreasing mortality.

Cancer risk seems stable in Danish patients with acromegaly, but further population-based studies including reference data from the background population are needed to properly elucidate the relationship between acromegaly and cancer risk.

## Data availability statement

The raw data supporting the conclusions of this article will be made available by the authors, without undue reservation.

## Ethics statement

Ethical approval was not required for the studies involving humans because the research did not involve direct experiments/sampling from human subjects. The studies were conducted in accordance with the local legislation and institutional requirements. The human samples used in this study were acquired from a by- product of routine care or industry. Written informed consent to participate in this study was not required from the participants or the participants’ legal guardians/next of kin in accordance with the national legislation and the institutional requirements.

## Author contributions

CR: Writing – original draft, Writing – review & editing, Conceptualization, Data curation, Formal Analysis, Investigation, Project administration, Visualization. MC-S: Writing – review & editing. EN: Writing – review & editing. MSA: Writing – review & editing. CF: Writing – review & editing. MK: Writing – review & editing. MA: Writing – review & editing. NB: Methodology, Visualization, Writing – review & editing. JJ: Writing – review & editing. JD: Conceptualization, Funding acquisition, Supervision, Writing – review & editing.
